# Hepatic stearoyl-CoA desaturase 1 suppresses diet-induced hyperphagia and hepatic lipid accumulation through PPARα activation and ketone body signaling

**DOI:** 10.1016/j.jbc.2026.113515

**Published:** 2026-09-22

**Authors:** Kun Jia, Yang Liu, Yang Zhang, Lina Bai, Lei Zhang, Fengjun Xie, Xiaojun Yan, Jilin Xu, Kai Liao

**Affiliations:** 1 School of Marine Sciences, Ningbo University, Ningbo, China; 2 National Engineering Research Center of Marine Facilities Aquaculture, Zhejiang Ocean University, Zhoushan, China

**Keywords:** SCD1, appetite regulation, liver–brain axis, pparα, ketogenesis, zebrafish

## Abstract

Stearoyl-CoA desaturase 1 (SCD1) is a key enzyme in fatty acid desaturation with a well-established role in lipid metabolism, but its function in appetite regulation remains insufficiently understood. Here, we investigated whether hepatic SCD1 regulates high-fat diet (HFD)-induced lipid accumulation and feeding behavior through liver-to-brain metabolic signaling. Long-term HFD feeding suppressed hepatic *scd1* expression in black seabream and zebrafish despite progressive hepatic lipid accumulation. Using liver-specific *scd1*-overexpressing zebrafish and peroxisome proliferator-activated receptor α (PPARα)-deficient *scd1*-overexpressing zebrafish, we found that hepatic *scd1* overexpression attenuated HFD-induced lipid deposition and markedly suppressed food intake in both larval and adult zebrafish. These effects were accompanied by reduced *agouti*-*related peptide* expression and increased *cocaine*- *and amphetamine*-*regulated transcript* (*cart*) and *gonadotropin*-*releasing hormone 2* expression. Mechanistically, hepatic *scd1* overexpression increased palmitoylethanolamide and oleoylethanolamide levels, enhanced hepatic PPARα nuclear translocation, and activated hepatic fatty acid oxidation and ketogenesis programs. Genetic ablation of PPARα weakened the lipid-lowering effect of *scd1* overexpression and partially restored orexigenic signaling. Moreover, hepatic *scd1* overexpression increased hepatic *3*-*hydroxy*-*3*-*methylglutaryl*-*CoA synthase 2* (*hmgcs2*) mRNA expression and elevated β-hydroxybutyrate and acetoacetate levels in both the liver and brain, accompanied by reduced brain AMP-activated protein kinase α phosphorylation. Together, these findings identify hepatic SCD1 as a metabolic regulator that suppresses HFD-induced hyperphagia and lipid accumulation, involving lipid-derived PPARα signaling and ketone body-mediated brain energy sensing.

Stearoyl-CoA desaturase 1 (SCD1), also known as Δ9 desaturase, is a key endoplasmic reticulum-localized enzyme that catalyzes the conversion of saturated fatty acids, such as palmitate and stearate, into their corresponding monounsaturated fatty acids (MUFAs), including palmitoleate and oleate (1). As a rate-limiting enzyme in *de novo* lipogenesis, SCD1 is critically involved in the maintenance of lipid homeostasis and the regulation of energy metabolism and body weight (2, 3). In mammals, SCD1 is highly expressed in metabolically active tissues, particularly the liver and adipose tissue, and its expression is strongly influenced by nutritional status, including high-carbohydrate and high-cholesterol diets (4, 5). Whole-body deletion of SCD1 results in a lean phenotype and protects against diet-induced obesity and insulin resistance, whereas tissue-specific manipulation of SCD1 produces distinct metabolic outcomes depending on the tissue context and dietary conditions (6, 7, 8, 9, 10, 11, 12, 13). In skeletal muscle, SCD1 overexpression has been reported to enhance metabolic capacity, fatty acid β-oxidation, and exercise performance, but may also increase susceptibility to lipid accumulation and insulin resistance under HFD conditions (14, 15). These findings indicate that SCD1 is not merely a lipogenic enzyme, but rather a context-dependent regulator of systemic lipid and energy metabolism.

In addition to its established role in lipid metabolism, emerging evidence suggests that SCD1 may also be involved in appetite regulation. This link is partly supported by the production of oleic acid, a major enzymatic product of SCD1 activity, which can serve as a precursor for oleoylethanolamide (OEA), an oleic acid-derived N-acylethanolamine with well-characterized anorexigenic effects mediated largely through peroxisome proliferator-activated receptor α (PPARα) activation (16). Consistently, stearoylethanolamide administration has been reported to reduce food intake in mice (17). Moreover, leptin treatment in ob/ob mice modulates hepatic scd1 expression and markedly suppresses SCD1 enzymatic activity, further suggesting a potential association between SCD1-dependent lipid metabolism and appetite-related endocrine signaling (18). Oleic acid itself has also been shown to act as a neurotrophic signal involved in neuronal migration, neurite outgrowth, and synapse formation during brain development (19). In fish, SCD1 has likewise been implicated in appetite control, as whole-body SCD1 deficiency in zebrafish has been reported to significantly suppress food intake (20). Nevertheless, systematic investigations into the role of SCD1 in appetite control and its underlying molecular mechanisms are still lacking.

High-fat diet (HFD) exposure has been shown to induce hepatic lipid accumulation and metabolic dysregulation in fish, thereby impairing organismal health and disrupting appetite regulation (21, 22). Similar to mammals, appetite regulation in teleosts is mainly coordinated by the hypothalamus and associated brain regions, where orexigenic neuropeptide Y/agouti-related peptide (NPY/AgRP) neurons and anorexigenic proopiomelanocortin/cocaine- and amphetamine-regulated transcript (POMC/CART) neurons integrate peripheral nutritional and metabolic signals (23, 24). However, whether hepatic lipid metabolic enzymes can communicate with the central appetite-regulatory network through liver-to-brain metabolic signaling remains poorly understood. Given the central role of SCD1 in hepatic MUFA production and the anorexigenic properties of oleic acid-derived lipid mediators such as OEA, hepatic SCD1 may represent a key metabolic node linking liver lipid homeostasis to central appetite control under HFD conditions.

Zebrafish (*Danio rerio*) have been widely established as a versatile model organism for studying lipid metabolism due to their rapid development, ease of maintenance, high reproductive capacity, well-annotated genome, and strong evolutionary conservation with other teleosts, together with the availability of advanced genetic manipulation techniques (25, 26). In the present study, zebrafish models combined with gene editing and liver-specific genetic manipulation were used to investigate whether hepatic SCD1 coordinates HFD-induced lipid accumulation and appetite regulation through liver-to-brain metabolic signaling. Our study aimed to clarify the role of hepatic SCD1 in regulating lipid-derived PPARα signaling, ketone body production, brain AMPK activity, and appetite-related neuropeptide expression, thereby providing new insight into the metabolic communication between hepatic lipid metabolism and feeding control.

## Results

### HFD induces SCD1 dysregulation accompanied by hepatic lipid deposition in fish

After 8 weeks of feeding, hepatic *scd1* mRNA expression was significantly decreased in juvenile black seabream fed HFD compared with the ND group (Fig. 1*A*), whereas a pronounced accumulation of lipid droplets was observed in the liver (Fig. 1*B*). Data on dietary composition and growth performance have been described previously (27). In zebrafish, hepatic *scd1* mRNA expression was significantly upregulated after 1 week of HFD feeding compared with the ND group. However, with prolonged feeding, *scd1* expression was progressively and significantly downregulated at 2 and 4 weeks (Fig. 1*C*). After 4 weeks, the ratio of C18:1 to C18:0 in both liver and muscle tissues was significantly lower in the HFD group than in the ND group (Fig. 1*D*). Throughout the experimental period, hepatic lipid levels, including triglycerides (TG), total cholesterol (TC), low-density lipoprotein cholesterol (LDL-C), and non-esterified fatty acid (NEFA), were consistently elevated in the HFD group compared with the ND group, with a progressive increase in accumulation over time (Fig. 1, *E*–*H*). In contrast, the expression patterns of other key lipogenic genes did not mirror that of *scd1*. Hepatic *fasn* mRNA level was consistently and significantly elevated in the HFD group (Fig. 1, *I–L*). In comparison, *acc1* expression was significantly increased only after 1 week of feeding, whereas *srebp1* expression showed a significant upregulation only at 4 weeks (Fig. 1, *I–L*). No significant differences were observed in *pparg* mRNA levels throughout the experimental period (Fig. 1, *I–L*). SCD1 proteins are highly conserved across most teleost species, exhibiting sequence homology exceeding 96% (Fig. S1*A*). Notably, amino acid sequences within the transmembrane regions and catalytic domains are largely conserved, indicating strong structural and functional preservation (Fig. S1*B*). Collectively, these results indicate that, in teleosts, hepatic *scd1* expression exhibits a time-dependent and distinct downregulation in response to prolonged HFD feeding, while lipid accumulation progressively increases.

**Figure 1:**
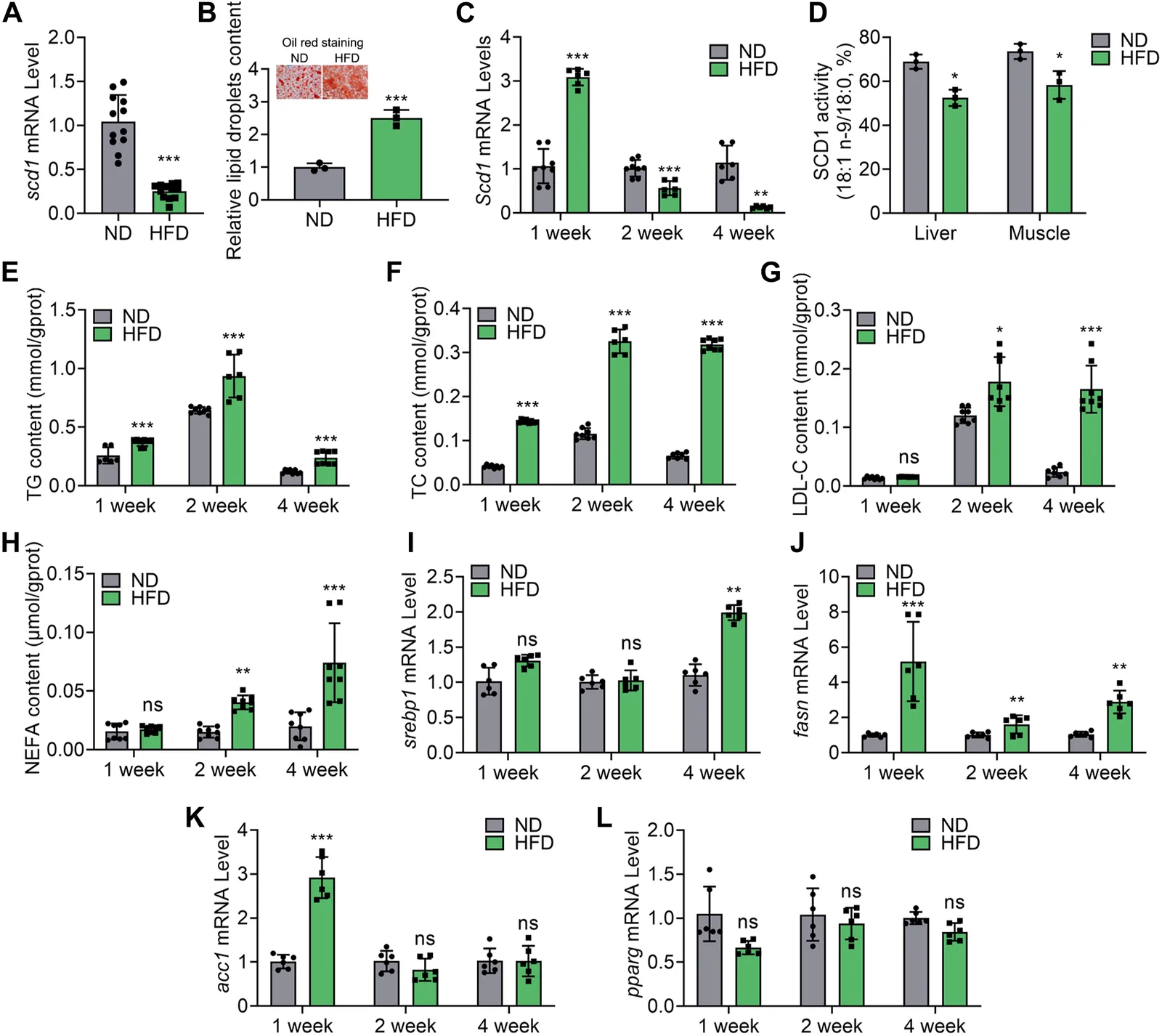
**The changes of *scd1* expression and lipid deposition in the liver of fish fed HFD.***A*, hepatic *scd1* mRNA expression levels in *black seabream*. *β-actin* as the internal reference gene. *B*, *Oil Red* O staining of the liver and quantification of lipid droplet accumulation in *black seabream*. *C*, hepatic *scd1* mRNA expression levels in zebrafish after 1, 2, and 4 weeks of feeding under ND and HFD conditions. *rps11* as the internal reference gene. *D*, ratio of C18:1 n-9 to C18:0 in the liver and muscle of zebrafish after 4 weeks of ND or HFD feeding. *E*–*H*, hepatic levels of TG (*E*), TC (*F*), LDL-C (*G*), and non-esterified fatty acid (NEFA) (*H*) in zebrafish under ND and HFD conditions after 1 week, 2 weeks, and 4 weeks of feeding. *I*–*L*, mRNA expression levels of key lipogenic genes (*srebp1*, *fasn*, *acc1*, and *pparg*) in the liver of zebrafish under ND and HFD conditions after 1 week (*I*), 2 weeks (*J*), and 4 weeks (*L*) of feeding. Gene expression was normalized to *rps11*. HFD, high-fat diet; ND, normal diet; PPARg, peroxisome proliferator-activated receptor gamma; SCD1, stearoyl-CoA desaturase 1.

### Liver-specific overexpression of *scd1* reduces lipid deposition in zebrafish

To further elucidate the role of SCD1 in HFD-induced lipid accumulation, we generated transgenic zebrafish with liver-specific overexpression of *scd1* (*scd1*^+/+^). The structure of the *scd1* overexpression construct is shown in Figure 2*A*. In this construct, the liver-specific promoter *lfabp10* was used to drive the expression of enhanced green fluorescent protein (EGFP) and *scd1*. A P2A peptide sequence was inserted between EGFP and *scd1* to enable coexpression while minimizing translational interference. Liver-specific overexpression of *scd1* did not affect normal development in zebrafish, and EGFP fluorescence was specifically detected in the liver, confirming successful tissue-specific expression (Fig. 2*B*). Whole-mount *in situ* hybridization revealed a marked increase in *scd1*^+/+^ zebrafish larvae compared with WT (Fig. 2*C*). In adult fish, hepatic *scd1* expression was elevated by approximately 200-fold in *scd1*^+/+^ zebrafish (Fig. 2*D*). Moreover, the expression of hepatic SCD1 protein was significantly elevated in *scd1*^+/+^ zebrafish (Fig. 2*E*). The EGFP was clearly detected in *scd1*^+/+^ zebrafish, and the observed band size was consistent with the expected molecular weight (25-35 KDa), indicating intact EGFP protein expression from the transgenic cassette (Fig. S2*A*). These results confirm the successful establishment of liver-specific *scd1* overexpression in zebrafish.

**Figure 2:**
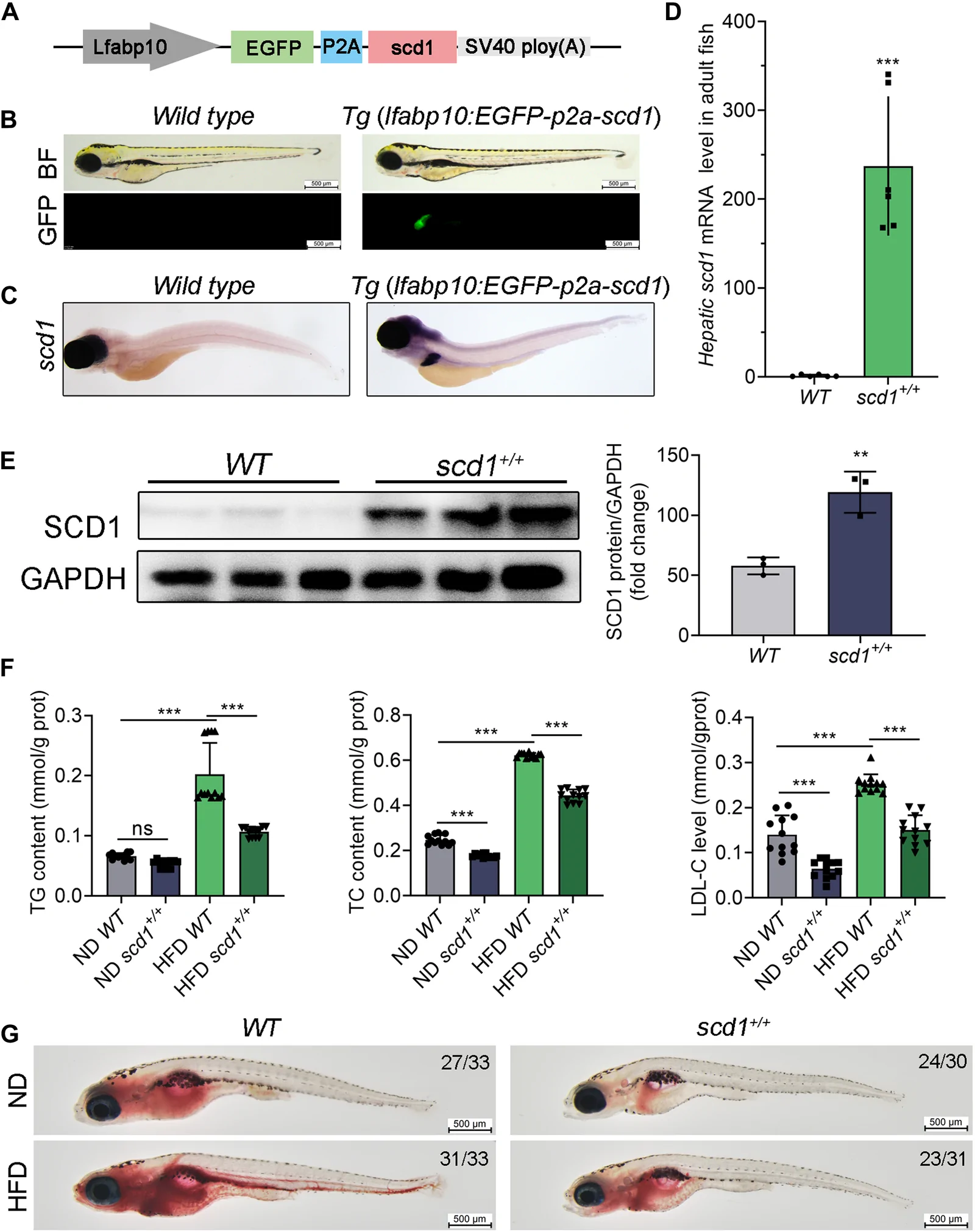
**Effects of liver-specific *scd1* overexpression on lipid accumulation after 14 days of larval feeding.***A*, schematic diagram of the transgenic construct. *B*, bright-field (BF) and fluorescence (GFP) images of 6 days postfertilization (dpf) larvae from WT and transgenic Tg (*lfabp10*:EGFP-P2A-*scd1*) zebrafish. The scale bar represents 500 μm. *C*, whole-mount *in situ* hybridization showing *scd1* expression in zebrafish larvae. *D*, hepatic *scd1* mRNA expression levels in adult zebrafish, normalized to *rps11* as the internal reference gene. *E*, the SCD1 and GAPDH immunoblots and quantitative analysis of the liver in *WT* and *scd1^+/+^* zebrafish using Western blotting. *F*, levels of TG, TC, and LDL-C in zebrafish larvae. *G*, representative *oil red* O staining images of zebrafish larvae. Numbers in the *upper right corner* indicate the proportion of individuals exhibiting the corresponding phenotype. The scale bar represents 500 μm. SCD1, stearoyl-CoA desaturase 1.

A 7-days larval feeding experiment was performed. Liver-specific *scd1* overexpression did not affect TG or TC levels in ND group but significantly reduced LDL-C (Fig. S2*B*). Under HFD conditions, *scd1* overexpression decreased TC and LDL-C levels while increasing TG (Fig. S2*B*). Oil Red O staining further confirmed a marked reduction in lipid deposition in *scd1*^+/+^ larvae compared with *WT* under both dietary conditions (Fig. S2*C*). Subsequently, a 14-days feeding experiment was subsequently performed. Liver-specific *scd1* overexpression did not affect TG levels under ND conditions but significantly reduced TC and LDL-C (Fig. 2*F*). Under HFD conditions, *scd1* overexpression markedly decreased all lipid-related parameters (Fig. 2*F*). Oil red O staining further confirmed reduced lipid accumulation in *scd1*^+/+^ larvae under both dietary conditions (Fig. 2*G*). These results suggest that prolonged *scd1* overexpression exerts a stronger protective effect against HFD-induced lipid accumulation than short-term intervention.

In parallel, a 4-weeks feeding experiment in adult zebrafish was further conducted. Growth performances, including weight gain and body length, were significantly reduced under both ND and HFD conditions in *scd1*^+/+^ fish compared to *WT* (Fig. 3, *A* and *C*), whereas viscerosomatic index (VSI) was significantly decreased only under HFD conditions (Fig. 3*B*). Under ND conditions, TG levels were increased, while TC and LDL-C levels were not affected (Fig. 3*B*). Under HFD conditions, all lipid-related parameters were markedly reduced (Fig. 3*D*). Oil red O staining further demonstrated that HFD-induced hepatic lipid accumulation was significantly attenuated in the *scd1*^+/+^ group compared to *WT* group (Fig. 3*E*). These results suggest that liver-specific *scd1* overexpression exerts a protective effect against HFD-induced obesity and hepatic steatosis in zebrafish.

**Figure 3:**
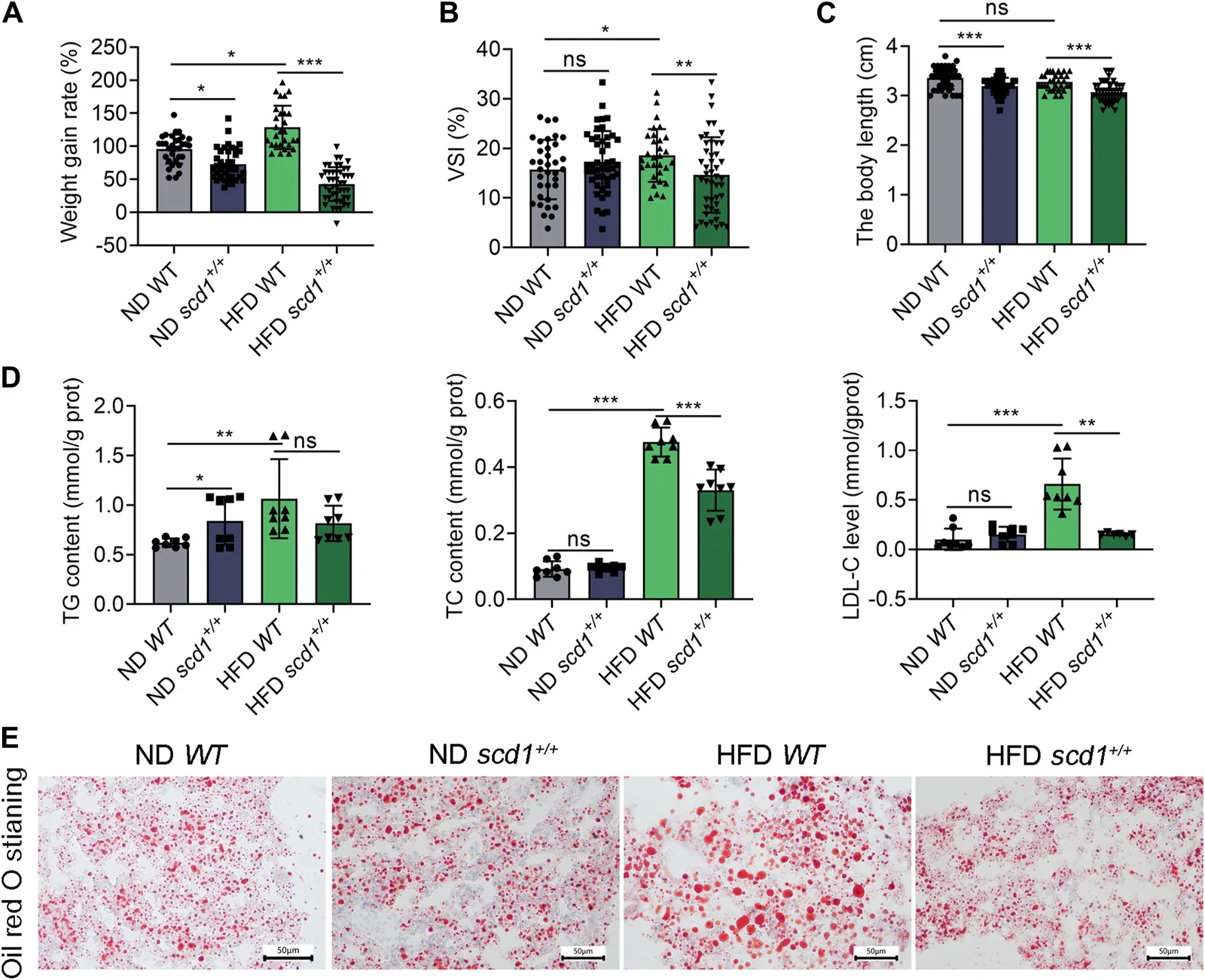
**Effects of liver-specific *scd1* overexpression on lipid accumulation after 4 weeks of adult zebrafish feeding.***A*–*C*, the physiological indices of adult male zebrafish after feeding for 4 weeks (*A*) the viscerosomatic index (VSI, %), (*B*) the weight gain rate (%), and (*C*) the body length (mm). *D*, the biochemical indicators in liver tissues of adult male zebrafish, including the contents of TG, TC, and LDL-C. *E*, the images of *Oil Red* O staining of liver tissues of adult male zebrafish. The scale bar represents 50 μm. SCD1, stearoyl-CoA desaturase 1.

### Liver-specific overexpression of *scd1* suppresses appetite in zebrafish

During the adult feeding experiment, a marked reduction in food intake was observed in the *scd1*^+/+^ group compared to *WT* group at approximately day 22, despite equal feed provision across groups (Fig. 4*A*). To more rigorously quantify feeding behavior, a pellet-based assay was implemented following established protocols (28), using particles with an average diameter of ∼1 μm (Fig. 4*B*). As shown in Figure 4, *C* and *D*, after 14 and 28 days of feeding, no significant difference in food intake was observed between ND and HFD conditions in the *WT* group, whereas a significant reduction was detected in the *scd1*^+/+^ group. Notably, *WT* fish displayed a time-dependent increase in food intake, whereas this increase was blunted in *scd1*^+/+^ fish, which maintained consistently lower intake levels.

**Figure 4:**
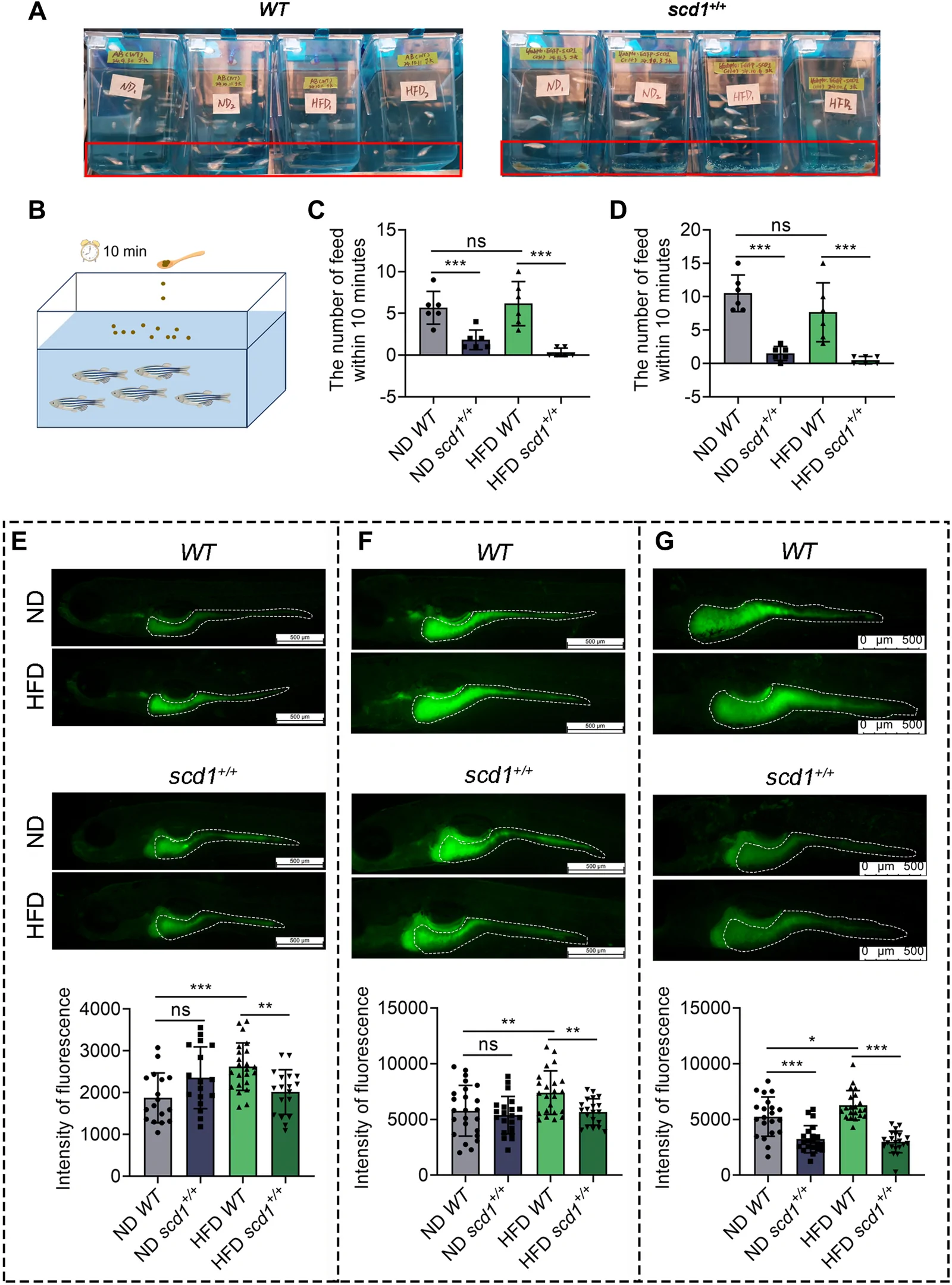
**Liver-specific *scd1* overexpression suppresses feeding behavior in zebrafish.***A*, bright-field images of adult zebrafish tanks 30 min after feeding. *B*, schematic illustration of the experimental design for quantifying food intake in adult zebrafish. *C* and *D*, quantification of food intake in adult zebrafish after 14 days of feeding (*C*) or 28 days of feeding (*D*). *E*, food intake after 1 day of normal feeding. *F*, food intake after 3 days of normal feeding. *G*, food intake after 7 days of normal feeding. *Left panel* shows BODIPY FL C16 *green* fluorescence imaging; *right panel* shows statistical analysis of *green* fluorescence intensity in larval intestine. The scale bar represents 500 μm. SCD1, stearoyl-CoA desaturase 1.

To further evaluate the effect of liver-specific *scd1* overexpression on feeding behavior, a BODIPY FL C16-labeled fatty acid assay was employed to quantify food intake in zebrafish larvae. After 1 day of feeding, a slight increase in food intake was observed in the *scd1*^+/+^group under ND condition, whereas it was significantly reduced under HFD condition (Fig. 4*E*). After 3 days of feeding, no significant difference was detected under ND conditions; however, whereas it was significantly reduced in *scd1*^+/+^ compared to *WT* under HFD condition (Fig. 4*F*). By day 7, food intake was significantly reduced in *scd1*^+/+^ compared to *WT* under both ND and HFD conditions, with a markedly stronger inhibitory effect compared with that observed at days 1 and 3 (Fig. 4*G*). These findings in larvae are consistent with those observed in adult fish and reveal a clear time-dependent trend. Collectively, these results demonstrate that liver-specific *scd1* overexpression significantly suppresses feeding behavior in zebrafish, particularly HFD-induced hyperphagia, and that this inhibitory effect becomes more pronounced with prolonged feeding.

Alterations in food intake may be associated with changes in the expression of appetite-regulating genes. To explore this possibility, the mRNA levels of key appetite-related factors were measured in the brains of adult zebrafish and in larvae. In adult brains, the expression of the orexigenic gene *agrp* was significantly reduced in the *scd1*^+/+^ group, while no significant changes were observed in other orexigenic factors, including *cnr1*, *galn*, *ghsra*, *npy*, *hcrt*, and *pmch* (Fig. 5*A*). In contrast, the expressions of four key anorexigenic genes (*cart1*, *cart4*, *gnrh2*, and *pyy*) were significantly upregulated in the *scd1*^+/+^ group compared with WT (Fig. 5*B*). A similar pattern was observed in larvae. Among orexigenic genes, only *agrp* expression was significantly decreased in the *scd1*^+/+^ group (Fig. 5*C*). Meanwhile, the expressions of anorexigenic genes (*cart1*, *cart3*, and *gnrh2*) were significantly elevated compared with *WT* larvae (Fig. 5*D*). Collectively, these results suggest that the suppressive effect of liver-specific *scd1* overexpression on feeding behavior in zebrafish may be mediated by downregulation of the orexigenic factor *agrp* and upregulation of anorexigenic factors, including *cart* and *gnrh2*.

**Figure 5:**
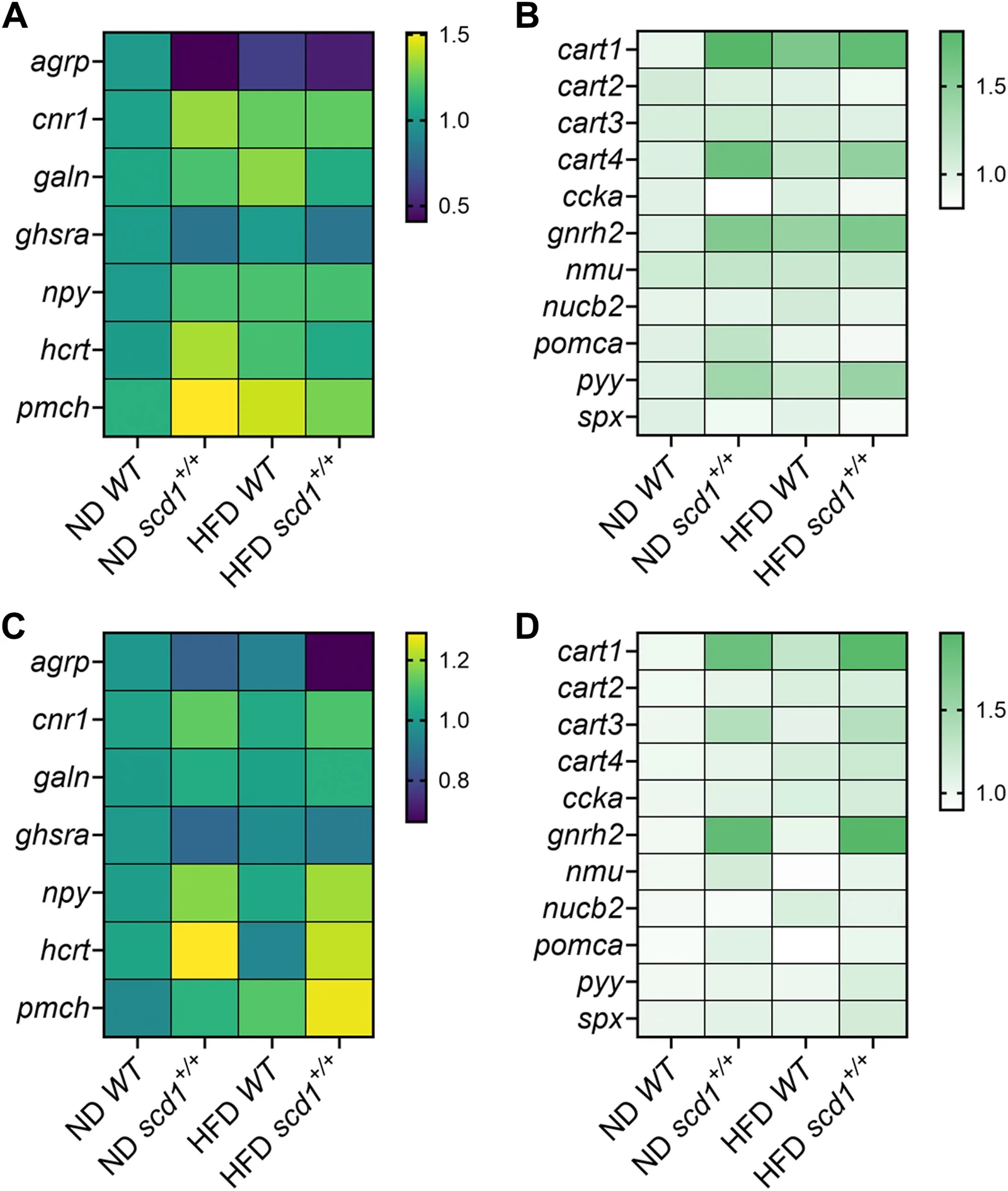
**Effects of hepatic *scd1* overexpression on the expression of appetite-related genes in zebrafish.***A*, mRNA expression of orexigenic factors (*agrp*, *cnr1*, *galn*, *ghsra*, *npy*, *hcrt*, and *pmch*) in the brain of adult zebrafish. *B*, mRNA expression of anorexigenic factors (*cart1*, *cart2*, *cart3*, *cart4*, *ccka*, *gnrh2*, *nmu*, *nucb2*, *pomca*, *pyy*, and *spx*) in the brain of adult zebrafish. *C*, mRNA expression of orexigenic factors in larvae. *D*, mRNA expression of anorexigenic factors in larvae. All genes were normalized to *β-actin* mRNA levels as an internal control. SCD1, stearoyl-CoA desaturase 1.

### PPARα mediates SCD1-dependent regulation of lipid metabolism and appetite

KEGG enrichment analysis of hepatic transcriptomes identified the PPAR signaling pathway, a key pathway regulating lipid metabolism, as significantly enriched in the HFD group compared with ND (Fig. 6*A*), the data were from our previous report (29). Given the established role of PPARα (a ligand-activated nuclear receptor involved in fatty acid metabolism) in appetite regulation (30, 31), its activation was further examined. Immunofluorescence analysis revealed enhanced nuclear localization of PPARα in the *scd1*^+/+^ group compared with *WT* group (Fig. 6*B*). Consistently, immunoblot analysis of nuclear and cytoplasmic fractions from primary hepatocytes confirmed a significant increase in nuclear PPARα levels in the *scd1*^+/+^ group compared with *WT* group without changes in cytoplasmic expression (Fig. 6*C*). Western blot analysis showed that GAPDH was absent or barely detectable whereas Lamin B1 was clearly detected in nuclear extracts, confirming sufficient nuclear purity for assessing nuclear PPARα levels (Fig. S3).

**Figure 6:**
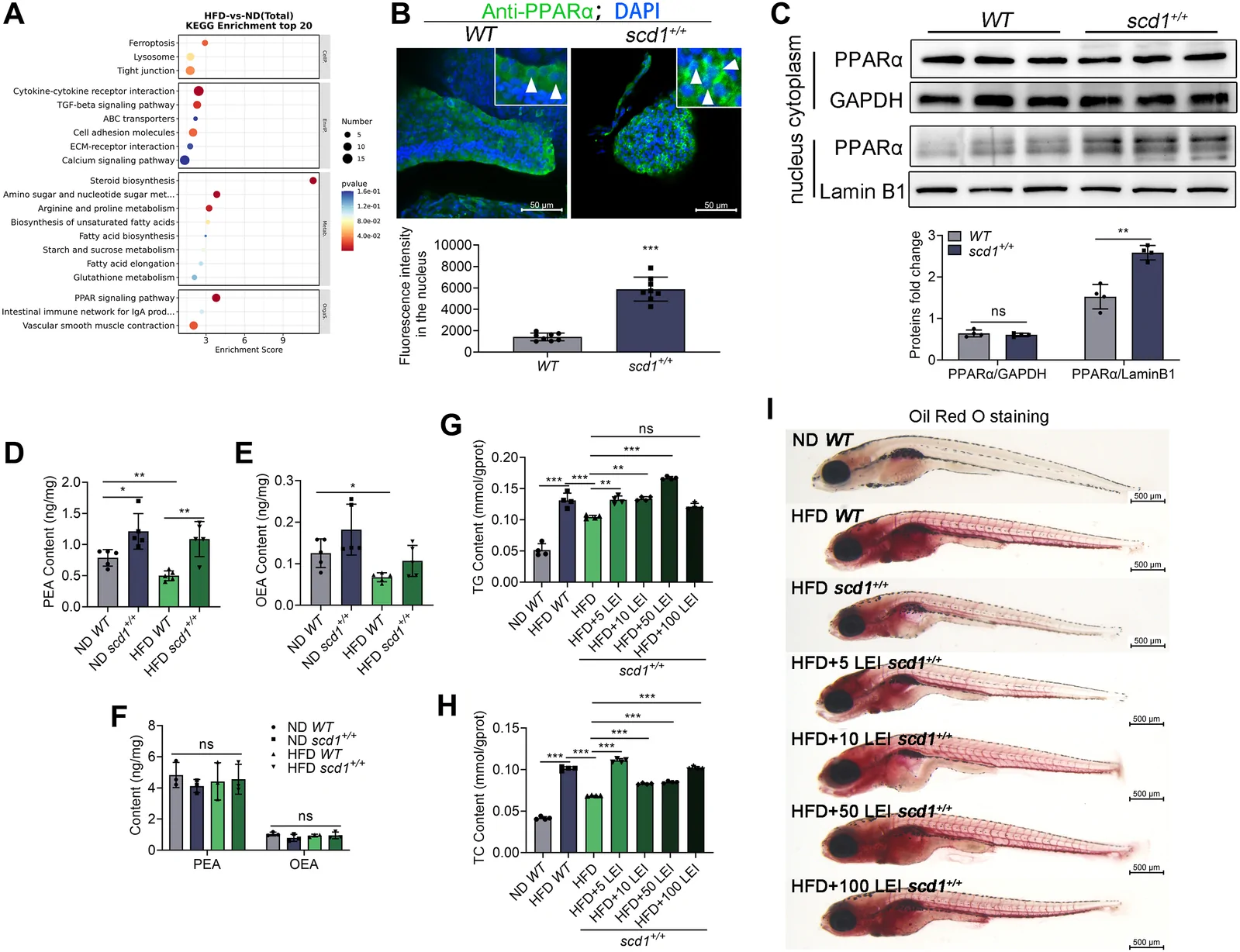
**Hepatic *scd1* overexpression enhances PPARα activation and hepatic PEA/OEA levels, while LEI-401 counteracts its lipid-lowering effects in HFD-fed zebrafish.***A*, KEGG enrichment analysis of liver transcriptome data from adult zebrafish in the HFD group *versus* the ND group. *B*, confocal images of immunofluorescence staining for PPARα protein in the liver of zebrafish larvae. *White triangles* indicate hepatocyte nuclei. The scale bar represents 50 μm. *C*, immunoblot analysis of nuclear translocation of PPARα protein in the livers of adult zebrafish. *D*, PEA content in the liver of adult zebrafish. *E*, OEA content in the liver of adult zebrafish. *F*, PEA and OEA contents in the brain of adult zebrafish. *G* and *H*, the TG (*G*) and TC (*H*) contents in the *WT* and *scd1*^+/+^ zebrafish larvae after feeding ND, HFD, HFD+5 mg/kg LEI401, HFD+10 mg/kg LEI401, HFD+50 mg/kg LEI401, and HFD+100 mg/kg LEI401 for 14 days *I*, whole-mount *Oil Red* O staining of zebrafish larvae. The scale bar represents 500 μm. HFD, high-fat diet; ND, normal diet; OEA, oleoylethanolamide; PEA, palmitoylethanolamide; PPARα, peroxisome proliferator-activated receptor α; SCD1, stearoyl-CoA desaturase 1.

To investigate the mechanism underlying SCD1-mediated activation of PPARα, the role of lipid-derived signaling molecules was examined. Given that SCD1 regulates the synthesis of MUFA and their derivatives, including palmitoylethanolamide (PEA) and OEA, these metabolites were quantified by HPLC–MS. Both standards and biological samples exhibited characteristic peaks at ∼10 min, confirming reliable detection (Fig. S4). Hepatic PEA levels were significantly increased in the *scd1*^+/+^ group and reduced by HFD feeding (Fig. 6*D*), while OEA showed a similar but less pronounced trend (Fig. 6*E*). No significant changes were observed in the brain (Fig. 6*F*). Subsequently, LEI-401, a specific inhibitor of N-acyl phosphatidylethanolamine phospholipase D (NAPE-PLD), a key enzyme involved in PEA and OEA biosynthesis, was used to further determine whether SCD1 functions by increasing PEA and OEA levels. In a 14-days feeding experiment, TG and TC levels were significantly increased in all HFD + LEI-401; *scd1*^+/+^ groups compared with the HFD *scd1*^+/+^ group (Fig. 6, *G* and *H*). Consistent with these biochemical findings, Oil Red O staining revealed a similar trend in lipid deposition (Fig. 6*I*). In addition, LEI-401 did not significantly increase TG levels in WT larvae (Fig. S5*A*), whereas 10 and 50 mg/kg LEI-401 significantly increased TC levels (Fig. S5*B*). However, LEI-401 did not produce a consistent regulatory pattern in the expression of the orexigenic gene *agrp* and the anorexigenic genes *cart1* and *gnrh2*. For example, *agrp* mRNA levels were significantly upregulated in the HFD + 5 mg/kg LEI-401; *scd1*^+/+^ group, but significantly downregulated in the HFD + 10 mg/kg LEI-401; *scd1*^+/+^ group, compared with the HFD *scd1*^+/+^ group (Fig. S5*C*). These findings suggest that SCD1 may activate PPARα through modulation of hepatic PEA and OEA levels.

To further validate PPARα as a key role of SCD1-mediated regulation of appetite, a PPARα KO zebrafish line was generated using the CRISPR–Cas9 system. The PPARα mutant carries a single-nucleotide deletion in exon 5, resulting in a frameshift and loss of the ligand-binding domain (Fig. 7, *A* and *B*). In a 14-days feeding experiment, TG and TC levels were significantly increased in the HFD *PPARα*^−/−^; *scd1*^+/+^ group compared with the HFD *scd1*^+/+^ group, but were not significantly different from those in the HFD *PPARα*^−/−^ group (Fig. 7*C*). Consistent with these biochemical findings, Oil Red O staining revealed a similar trend in lipid deposition (Fig. 7*D*). Moreover, PPARα KO restored orexigenic signaling, with increased *agrp* expression and decreased *cart1* and *gnrh2* mRNA levels (Fig. 7*E*). Overall, these findings suggest that PPARα is likely involved as a key intermediary in the regulation of appetite and lipid metabolism by SCD1.

**Figure 7:**
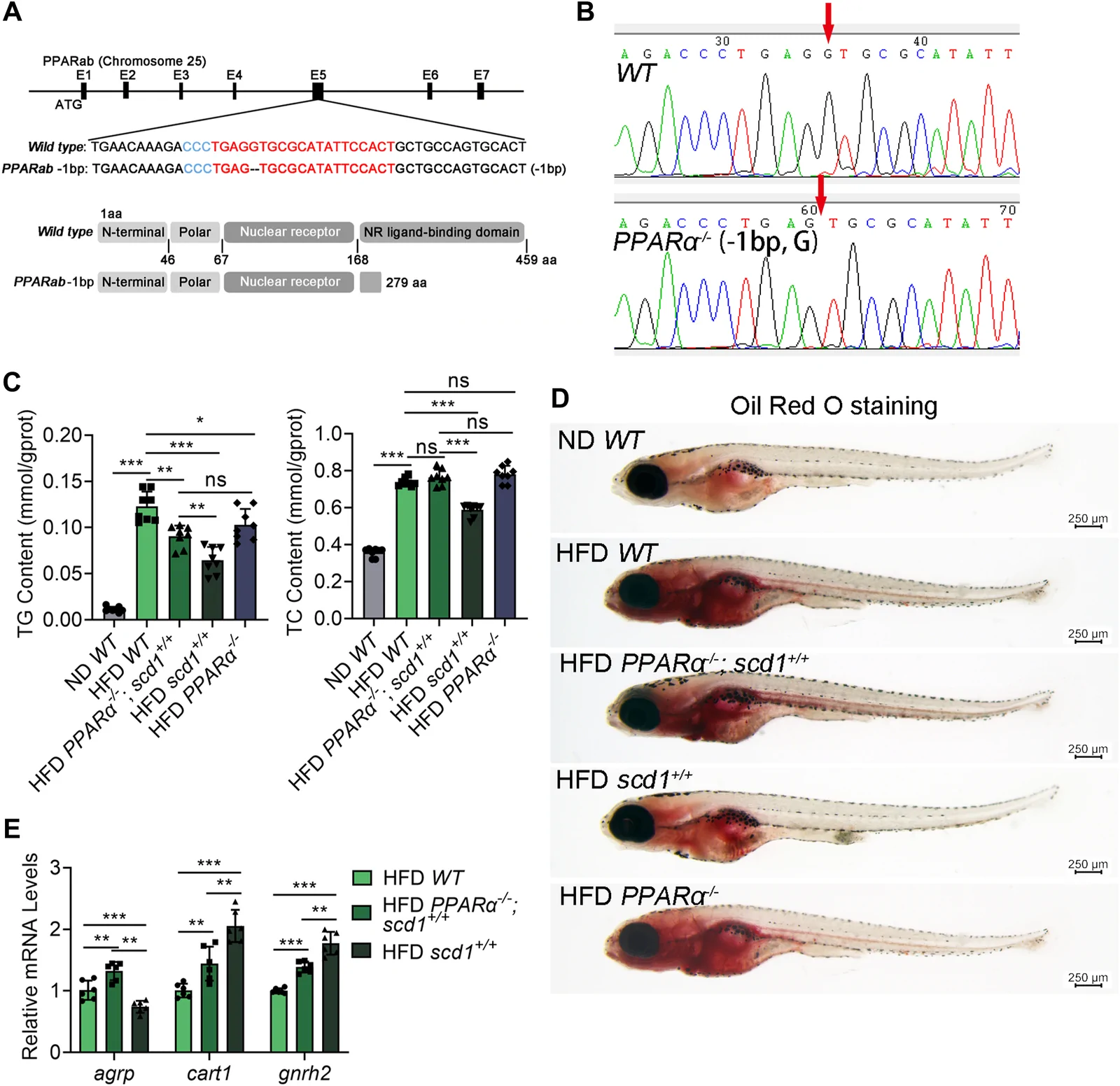
**Effect of PPARα KO on SCD1-mediated regulation of appetite and lipid metabolism.***A*, schematic diagram of the PPARα gene edited allele. Bases marked in *red* correspond to the CRISPR-Cas9 gRNA target sequence; bases marked in *blue* correspond to the NGG (GGG) PAM sequence. *B*, Sanger sequencing validation of and homozygous PPARα KO (*PPARα*^−/−^) zebrafish larvae. *C*, TG and TC levels in zebrafish larvae. *D*, whole-mount *Oil Red* O staining of zebrafish larvae. The scale bar represents 250 μm. *E*, mRNA expression levels of *agrp*, *cart*, and *gnrh2*. All values were normalized to *β-actin* as internal reference. PPARα, peroxisome proliferator-activated receptor α; SCD1, stearoyl-CoA desaturase 1.

### SCD1–PPARα signaling regulates appetite through ketone body–mediated hypothalamic energy sensing

To elucidate the downstream of SCD1-PPARα mediated regulation of appetite, the expression of PPARα target genes was examined in adult zebrafish. The expression levels of genes associated with fatty acid transport and β-oxidation (*acox1*, *cpt1a*, *ehhadh*, and *cd36*), as well as a rate-limiting ketogenic enzyme, 3-hydroxy-3-methylglutaryl-CoA synthase 2 (*hmgcs2*), were significantly upregulated in the liver of *scd1*^+/+^ fish compared with *WT* (Fig. 8*A*). In contrast, these genes were not differentially expressed in the brain; however, the secreted metabolic regulators fibroblast growth factor 21 (*fgf21*) and angiopoietin-like 4 (*angptl4*) were significantly elevated (Fig. 8*B*), suggesting tissue-specific downstream responses to PPARα activation. The NEFA level was not differentially in the liver of *WT* and *scd1*^+/+^ (Fig. S6).

**Figure 8:**
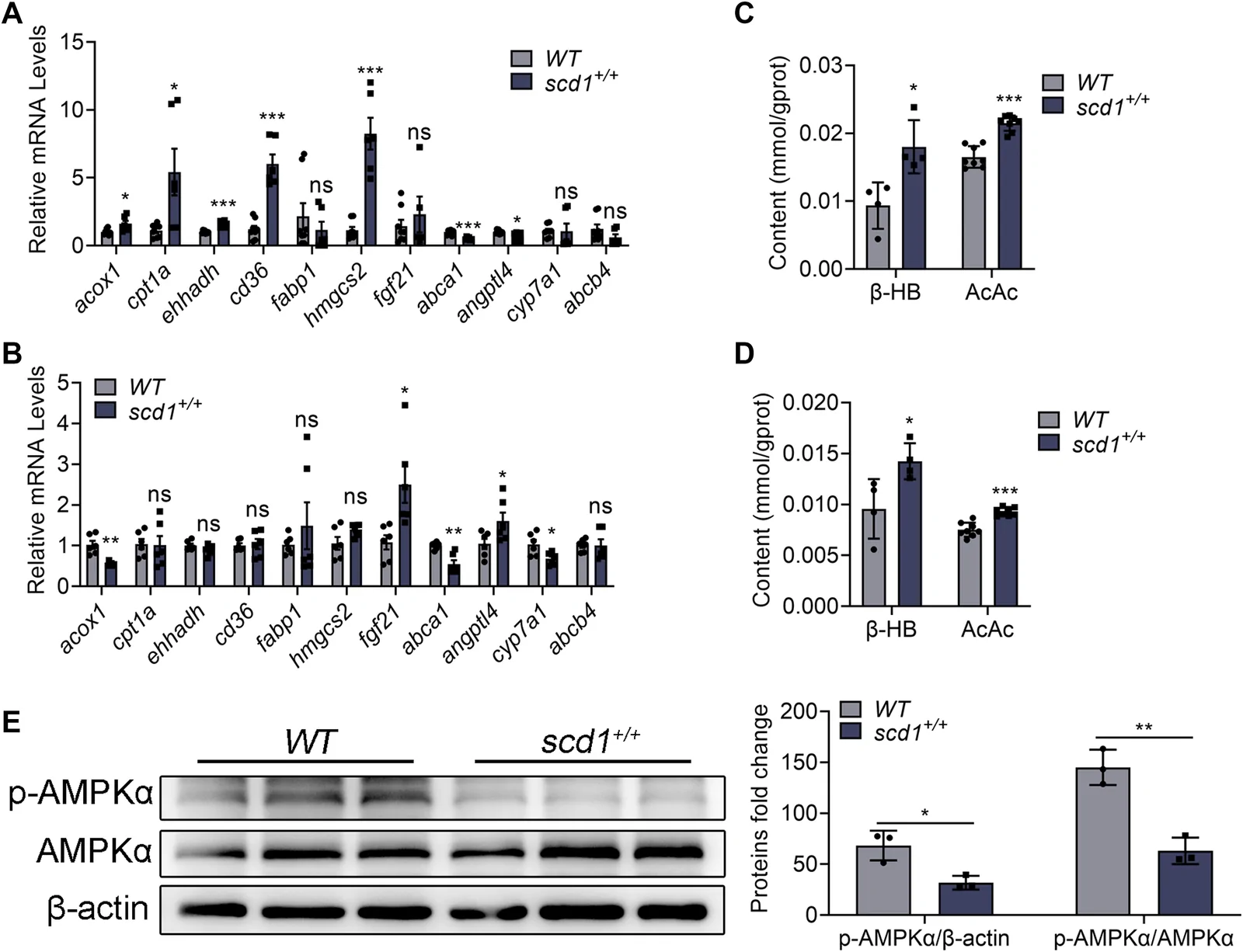
**Ketone body synthesis in the liver and brain of zebrafish and changes in energy-sensing signaling in the brain.***A*, hepatic mRNA expression of PPARα target genes (*acox1*, *cpt1a*, *ehhadh*, *cd36*, *fabp1*, *hmgcs2*, *fgf21*, *abca1*, *angptl4*, *cyp7a1*, and *abcb4*). *B*, brain mRNA expression of PPARα target genes (*acox1*, *cpt1a*, *ehhadh*, *cd36*, *fabp1*, *hmgcs2*, *fgf21*, *abca1*, *angptl4*, *cyp7a1*, and *abcb4*). The mRNA level of *Rps11* was used as an internal control. *C*, hepatic ketone body contents (β-HB and AcAc). *D*, brain ketone body contents (β-HB and AcAc). *E*, immunoblot analysis of AMPKα protein expression and its phosphorylation in the brain of adult zebrafish. β-HB, β-hydroxybutyrate; AcAc, acetoacetate; PPARα, peroxisome proliferator-activated receptor α.

Given the pronounced upregulation of *hmgcs2*, ketone body production was hypothesized to mediate SCD1–PPARα-dependent appetite regulation. Consistent with this hypothesis, levels of β-hydroxybutyrate (β-HB) and acetoacetate (AcAc) were significantly elevated in both the liver and brain of *scd1*^+/+^, with a greater increase observed in the liver (Fig. 8, *C* and D). Considering that ketone bodies readily cross the blood–brain barrier, these findings suggest a liver-to-brain metabolic signaling axis (32, 33). Furthermore, the phosphorylation level of AMP-activated protein kinase α (AMPKα), a key regulator of cellular energy sensing, was significantly reduced in the brains of *scd1*^+/+^ adult zebrafish compared with WT (Fig. 8*E*). Collectively, these results support a model in which SCD1–PPARα signaling modulates appetite *via* ketone body–hypothalamic energy-sensing mediated liver-to-brain communication.

## Discussion

Food intake and energy homeostasis in teleost fish are governed by highly complex regulatory networks, in which liver–brain axis signaling has been recognized as a central integrative pathway (34, 35). Although SCD1 has been implicated in appetite regulation, with its deficiency reported to suppress food intake (20), accumulating evidence also indicates that loss of SCD1 leads to obesity and hepatic steatosis in zebrafish (36, 37). These seemingly paradoxical findings highlight a critical gap in our understanding of SCD1 function in teleost and underscore the need to elucidate the molecular mechanisms by which SCD1 coordinates lipid metabolism and appetite, particularly under metabolic stress conditions such as obesity and fatty liver. In the present study, a zebrafish model was utilized to delineate a molecular framework underlying SCD1-mediated regulation of appetite in teleost fish. Liver-specific overexpression of *scd1* was shown to elevate the levels of PEA and OEA, thereby facilitating the activation of PPARα. Subsequent activation of PPARα promoted hepatic ketogenesis, and the resulting increase in circulating ketone bodies was transmitted to the hypothalamus, where it was integrated by AMPK-dependent energy-sensing pathways. This liver-to-brain signaling cascade led to suppression of the orexigenic neuropeptide *agrp* and concomitant induction of anorexigenic neuropeptides *gnrh2* and *cart*, ultimately attenuating HFD-induced hyperphagia.

In this study, long-term HFD feeding was found to markedly suppress hepatic *scd1* expression in both black seabream and zebrafish. This pattern appears to be conserved across multiple teleost species, as prolonged HFD exposure has also been reported to reduce hepatic *scd1* expression and enzymatic activity in common carp and blunt snout bream (38, 39). In addition, olive oil-based high-fat feeding decreases hepatic *scd1* expression in rainbow trout (40), while lysine-supplemented HFD similarly reduces SCD1 enzymatic activity in African catfish (41). Collectively, these findings suggest that downregulation of SCD1 in response to long-term lipid overload may represent a conserved metabolic adaptation in teleost fish. In mammals, both dietary and genetic models of obesity are characterized by elevated SCD1 expression, which promotes lipid synthesis and contributes to hepatic steatosis (42, 43), in clear contrast to the regulatory patterns observed in teleost fish. Notably, whole-body or liver-specific *scd1* deficiency in mice confers robust resistance to HFD or carbohydrate -induced obesity and fatty liver (7, 44), whereas in zebrafish, loss of SCD1 leads to dysregulated lipid accumulation and distribution, and can even result in obesity and hepatic steatosis (36, 37). This apparent functional divergence may be partially explained by the lineage-specific loss of SCD5 in teleost, raising the possibility that teleost SCD1 has retained or functionally integrated roles that, in mammals, are distributed across multiple SCD paralogs (45). In the present study, SCD1 proteins were found to be highly conserved among teleost species, with sequence identities exceeding 96%, whereas substantially lower homology was observed relative to mammals, suggesting that SCD1 function has undergone significant evolutionary divergence across vertebrate lineages. To further elucidate its functional role, transgenic zebrafish with liver-specific overexpression of *scd1* (*scd1*^+/+^) were generated. Comparative analyses under both ND and HFD conditions revealed that hepatic *scd1* overexpression markedly reduced lipid accumulation, particularly TC and LDL-C. Consistent with these findings, activation of SCD1 has been shown to enhance MUFA synthesis and alleviate HFD-induced metabolic and vascular dysfunction in mammals (46). Together, these results suggest that increased MUFA production may represent a conserved protective mechanism underlying the metabolic benefits of SCD1 activation. Thus, although zebrafish do not fully recapitulate mouse SCD1 biology, the presented research provides a useful complementary vertebrate model for examining species-specific roles of SCD1 in hepatic lipid metabolism under nutritional stress.

Under HFD conditions, the decrease in VSI was accompanied by reduced hepatic TG, TC, and LDL-C levels and attenuated Oil Red O staining in *scd1*^+/+^ fish. Because liver-specific *scd1* overexpression did not significantly alter VSI under ND conditions, the reduced VSI observed under HFD conditions is unlikely to reflect impaired visceral development caused by *scd1* overexpression itself. Instead, this pattern suggests that hepatic *scd1* overexpression reduces HFD-induced visceral/hepatic lipid burden and improves lipid metabolic status under nutritional stress. Nevertheless, because VSI is an indirect physiological index, it should be interpreted together with biochemical lipid measurements and histological staining rather than as an independent indicator of overall health.

Dysregulation of appetite has been recognized as a key factor exacerbating the adverse metabolic consequences of HFD exposure. Accumulating evidence indicates that SCD1 plays a role in the regulation of feeding behavior. In mammalian systems, reduced hepatic *scd1* expression has been associated with decreased food intake (17). Similarly, in zebrafish, whole-body deletion of *scd1* has been shown to markedly suppress feeding behavior and reduce body weight (20). These findings collectively suggest a conserved involvement of SCD1 in appetite regulation across vertebrate species. The results of the present study demonstrate that liver-specific overexpression of *scd1* markedly suppresses feeding behavior in zebrafish, with the magnitude of this effect progressively increasing with feeding duration. Because liver-specific *scd1* overexpression reduced food intake, its potential influence on growth and physiological status should be considered. Consistently, *scd1*^+/+^ fish showed reduced weight gain rate and body length under both ND and HFD conditions, indicating that appetite suppression was associated with decreased growth performance. Mechanistically, *scd1* overexpression is associated with significant downregulation of the orexigenic neuropeptide gene *agrp*, accompanied by upregulation of anorexigenic neuropeptide genes *cart* and *gnrh2*, indicating a coordinated shift toward appetite suppression at the neuroendocrine level. Notably, the present findings differ from previous reports demonstrating that whole-body deletion of *scd1* also results in reduced food intake (20), highlighting an apparent paradox in SCD1-mediated regulation of feeding behavior. From a metabolic perspective, both overexpression and deficiency of *scd1* may perturb the dynamic equilibrium of fatty acid desaturation and its bioactive derivatives, thereby influencing energy-sensing pathways and neuroendocrine circuits governing appetite. Consequently, disruption of SCD1 activity in either direction may converge on a similar phenotype of suppressed feeding. It should be noted that, in zebrafish, both whole-body deficiency and liver-specific overexpression of SCD1 have been shown to suppress feeding behavior, indicating that this bidirectional effect is not unique to SCD1. Similar phenotypic outcomes arising from both loss- and gain-of-function perturbations have been reported for other key regulatory genes, such as methyl-CpG-binding protein 2 (MECP2) (47) and *period* genes (48), which induce neurodevelopmental abnormalities or circadian rhythm disturbances when expression deviates from physiological levels. These findings collectively suggest that disruption of homeostatic gene expression, rather than the direction of change *per se*, may underlie convergent physiological and behavioral outcomes. Notably, the inhibitory effect of hepatic *scd1* overexpression on feeding observed in this study contrasts with mammalian evidence, in which reduced SCD1 expression is associated with decreased appetite (17). This divergence may reflect fundamental differences in hepatic lipid metabolic organization and nutrient utilization strategies between teleost fish and mammals, thereby highlighting the evolutionary plasticity of SCD1 function in energy homeostasis.

KEGG enrichment analysis of hepatic transcriptomic data (HFD *versus* ND) revealed significant enrichment of the PPAR signaling pathway. PPARs, including the α, γ, and β/δ isoforms, are ligand-activated nuclear receptors responsive to fatty acids and other lipid-derived molecules, and are known to play critical roles in energy metabolism and appetite regulation (32, 49). Within this pathway, activation of PPARα has been shown to be closely associated with SCD1 enzymatic activity and to play a key role in the regulation of feeding behavior (50, 51). Accordingly, subsequent analyses were focused on PPARα. Subsequently, liver-specific overexpression of *scd1* was found to significantly enhance PPARα activation and promote its nuclear translocation. Furthermore, genetic ablation of PPARα in the *scd1*^+^/^+^ background markedly attenuated the lipid-lowering effects and feeding regulation mediated by *scd1* overexpression in zebrafish. These findings indicate that PPARα functions as a critical intermediate mediator in SCD1-dependent regulation of appetite and lipid metabolism. It was further observed that liver-specific overexpression of *scd1* significantly increased the levels of PEA and OEA in the liver, while no significant changes were detected in the brain. In addition, treatment with LEI-401, an inhibitor of NAPE-PLD, which is a key rate-limiting enzyme in PEA/OEA biosynthesis (52), counteracted the lipid-lowering effect induced by liver-specific overexpression of *scd1*. PEA and OEA, as fatty acid-derived bioactive lipids, are recognized as potent endogenous ligands of PPARα (49, 53), and their biosynthesis is closely associated with SCD1 enzymatic activity. In mammals, upregulation of *scd1* has been reported to promote OEA production while suppressing PEA generation (1). In contrast, in the zebrafish model used in this study, hepatic *scd1* upregulation resulted in concomitant increases in both OEA and PEA levels. This species-specific divergence in N-acylethanolamine metabolism and PPARα activation patterns may underlie the distinct phenotypic outcomes of *scd1* deficiency, which manifests as leanness in mammals but obesity in zebrafish (7, 36, 37).

This study further found that activation of PPARα significantly upregulated the mRNA expression of *hmgcs2*. HMGCS2 is the rate-limiting enzyme in ketogenesis, catalyzing the condensation of acetyl-CoA and acetoacetyl-CoA derived from fatty acid β-oxidation to form 3-hydroxy-3-methylglutaryl-CoA (HMG-CoA); subsequently, HMG-CoA is cleaved to produce AcAc, which is further reduced to β-HB (50). Upregulation of *hmgcs2* expression was associated with enhanced ketone body production in the liver and elevated ketone body levels in the brain of zebrafish. And the elevated ketone body levels do not appear to be driven by changes in free fatty acid levels. Consistently, increased plasma ketone body levels have also been reported in *scd1*^−/−^ mice (7), suggesting a conserved link between SCD1 activity and systemic ketogenesis. Ketone bodies, including AcAc and β-HB, are transported *via* the circulation to extrahepatic tissues such as the brain and skeletal muscle, where they are oxidized to generate energy (51), and are also sensed by the AMPK complex in the brain (54). In the present study, a significant reduction in AMPKα phosphorylation was observed in the brains of *scd1*^+/+^ zebrafish, indicating that the AMPK signaling pathway perceives a state of energy sufficiency and integrates metabolic cues to suppress the expression of orexigenic factors while promoting the expression of anorexigenic factors.

In conclusion, this study demonstrates that HFD suppresses hepatic SCD1 expression and enzyme activity in teleost fish, while the liver-overexpression of *scd1* suppresses HFD-induced hyperphagia in zebrafish, thereby improving lipid accumulation. At the molecular level, *scd1* coordinates appetite regulation through a liver–brain axis-mediated inter-organ communication network, integrating hepatic SCD1–PEA/OEA–PPARα–ketone signaling with hypothalamic ketone–AMPK–appetite factor pathways.

## Experimental procedures

### Materials

PEA (CAS No.: 544-31-0, Cat. No.: HY-20685), OEA (CAS No.: 111-58-0, Cat. No.: HY-107542), and LEI-401 (LEI, CAS No.: 2393840-15-6, Cat. No.: HY-131181) were purchased from MedChemExpress. Oil red O powder (A600395) was obtained from Sangon Biotech. BODIPY FL C16 (D3821) was purchased from Thermo Fisher Scientific.

### Zebrafish and breeding

WT AB strain zebrafish were sourced from the China Zebrafish Resource Center (CZRC) and maintained in a recirculating aquaculture system. Fish were fed live brine shrimp two to three times per day to satiety. Water conditions were carefully controlled, with temperature maintained at 27 to 28 °C, conductivity at 500 to 550 μS/cm, and pH ranging from 7.0 to 7.5. A 14:10 h light–dark cycle was used throughout the study. All experimental procedures were approved by the Institutional Animal Care and Use Committee of Ningbo University.

Experimental diets were prepared as our previous report (25). LEI-401 powder was dissolved in DMSO to obtain a 100 mg/ml stock solution. AP100 feed, cholesterol powder, egg yolk powder, and the required amount of LEI-401 stock solution were combined in a 250 ml glass beaker according to the designated formulation. Ether was then added to the mixture at a volume of 30 ml, followed by incubation in a 50 °C water bath to facilitate homogeneous mixing. Once the ether had completely evaporated, the diet was spread evenly on a tray and dried at low temperature before use.

### Generation of liver-specific SCD1-overexpressing transgenic zebrafish

To generate liver-specific SCD1-overexpressing transgenic zebrafish, the coding sequence of *scd1* was amplified and cloned downstream of a liver-specific promoter (*lfabp10a*). The recombinant construct was inserted into a Tol2 transposon-based vector. The resulting plasmid, together with Tol2 transposase mRNA, was coinjected into one-cell stage zebrafish embryos. Injected embryos (F0) were raised to 6-day-old larvae and screened for germline transmission by fluorescence marker expression or PCR-based genotyping. Positive founders were crossed with WT fish to obtain F1 offspring, and stable transgenic lines were established through subsequent generations. Liver-specific overexpression of *scd1* was confirmed by quantitative PCR. The spatial specificity of transgene expression was further verified by fluorescence imaging or *in situ* hybridization. The sequence of primers used was detailed in the Table S1.

### Generation of PPARα mutant zebrafish

PPARα-deficient zebrafish were generated using the CRISPR/Cas9 genome editing system following previously established procedures (55). Briefly, guide RNAs (gRNAs) targeting *pparα* were synthesized *in vitro* using a T7 RNA polymerase transcription kit. The gRNAs (100 ng/μl) were mixed with EnGen Spy Cas9 NLS protein (M0646T, NEB) and coinjected into the animal pole of one-cell stage embryos derived from homozygous liver-specific SCD1-overexpressing transgenic zebrafish. At 72 h postfertilization (hpf), a subset of embryos was randomly collected for genotyping. Genomic DNA was extracted using a tissue/cell genomic DNA isolation kit, and the target region was amplified by PCR. Indel mutations were verified by Sanger sequencing. The sequences of gRNAs and PCR primers are provided in Table S1. Founder fish carrying the desired mutations were subsequently bred for multiple generations, and screening was performed to establish stable homozygous lines with both *pparα* deficiency and liver-specific SCD1 overexpression.

### Quantitative real-time PCR

Total RNA was extracted using a commercial kit (ER501-01-V2, TransGen Biotech, China) and quantified with a NanoDrop spectrophotometer. Approximately 1 μg of RNA was reverse-transcribed into cDNA using a reverse transcription kit (AG11728, Accurate Biotechnology, Hunan, China), which was stored at −20 °C. Quantitative PCR was performed using SYBR Green premix (AG11701, Accurate Biotechnology, Changsha, China) on a real-time PCR system (Q2000A, LongGene, Hangzhou, China). Primer sequences used in this study are listed in Table S1.

### Western blotting

Western blotting was performed as previously described (56). Briefly, Nuclear and cytoplasmic proteins were extracted using a commercial extraction kit (P0027, Beyotime Biotechnology) according to the manufacturer’s instructions. Total protein was prepared with RIPA lysis buffer (PC101, Epizyme) supplemented with protease and phosphatase inhibitors (Invitrogen). Protein concentration was determined by BCA assay. Samples were denatured after mixing with loading buffer and subjected to SDS-PAGE, followed by transfer onto PVDF membranes. Membranes were blocked with 5% nonfat milk and incubated with primary and secondary antibodies (1:10,000 dilution) at 4 °C overnight. Protein signals were detected using a chemiluminescence imaging system (UVP ChemStudio, Analytik Jena) and quantified with ImageJ software. The primary antibodies lists: anti-PPARα (1:1000, Cat No.:66826-1, Proteintech), anti-AMPKα (1:1000, #2532, Cell Signaling Technology), anti-p-AMPKα (1:1000, #2535, Cell Signaling Technology), anti-SCD1 (1:1000, HA601180, HUABIO), anti-GAPDH (1:3000, AF0006, Beyotime Biotechnology), anti-β-actin (1:3000, AF0003, Beyotime Biotechnology), anti-EGFP (1:2500, AF1483, Beyotime Biotechnology), and anti-Lamin B1 (1:20,000, AF1408, Beyotime Biotechnology).

### Oil red O staining, lipid content, and enzyme activity assays

Lipid accumulation in larvae and liver tissues were assessed by Oil Red O staining and analysis as previously described (25). Briefly, samples were fixed, stained with Oil Red O solution, and imaged under a light microscope. The relative lipid droplet content in liver sections was quantified from Oil Red O-stained images using ImageJ. Briefly, the Oil Red O-positive staining signal in each liver section was converted into grayscale intensity and quantified under the same thresholding and imaging conditions. The values were then normalized to the ND group and expressed as relative lipid droplet content.

Total lipid content, including triglycerides (TG), total-cholesterol (TC), low-density lipoprotein cholesterol (LDL-C), and NEFA, was measured using commercial assay kits according to the manufacturers’ instructions.

### Whole-mount *in situ* hybridization and immunofluorescence staining

Whole-mount *in situ* hybridization (WISH) and immunofluorescence staining were performed as previously described (57). DIG-labeled antisense RNA probes for *scd1* were synthesized from embryonic cDNA-derived templates by *in vitro* transcription using the probe synthesis system (Roche) and stored at −80 °C. Whole-mount *in situ* hybridization was carried out using standard procedures, including larvae fixation, methanol dehydration, proteinase K permeabilization, overnight hybridization, anti-DIG-AP incubation, and chromogenic detection. After final washes, samples were imaged using a stereomicroscope (Zeiss SteREO Discovery.V20).

Immunofluorescence staining was performed on larvae following standard procedures. Briefly, larvae were fixed overnight in 4% PFA, permeabilized, and blocked before incubation with primary antibodies at 4 °C overnight, followed by fluorescence-conjugated secondary antibodies. After final washes, samples were imaged using a laser-scanning confocal microscope (Leica TCS SP8).

### Food intake assay

Larval feeding assay: Food intake in larvae was quantified using a fluorescent tracer-based method. Briefly, a 5% egg yolk emulsion was prepared and supplemented with BODIPY FL C16 to a final concentration of 6.4 μM. After 14 days of feeding with either a ND or HFD, larvae were transferred to petri dishes, and the medium was replaced with 5 ml of the BODIPY-containing emulsion. Larvae were allowed to feed for 2 to 3 h, followed by three washes to remove excess dye. Fluorescence images were acquired using a fluorescence microscope, and fluorescence intensity was quantified using ImageJ software as an indicator of food intake.

Adult feeding assay: Food intake in adult zebrafish was assessed by particle counting. At the designated time points (after 2 or 4 weeks of feeding), five size-matched fish per group were transferred to small tanks containing ∼5 cm of water and allowed to acclimate for 20 to 30 min. Subsequently, a fixed number of food pellets (n = 50) was provided, and the remaining pellets were counted after 10 min. Food intake was calculated based on the difference between the initial and remaining pellet numbers.

### Determination of PEA and OEA

PEA and OEA were extracted using a chloroform–methanol–water-based lipid extraction method. Briefly, freeze-dried samples were homogenized in chloroform:methanol: water containing BHT, followed by repeated extraction, phase separation with chloroform and saturated saline, and collection of the lower organic phase. After evaporation under nitrogen, residues were reconstituted in dichloromethane: methanol (3:1, v/v) for analysis.

Quantification of PEA and OEA was performed by HPLC–MS/MS using a C18 column coupled to an API 4000 triple quadrupole mass spectrometer operating in positive ion mode. Separation was achieved with a gradient elution of ammonium acetate-containing aqueous and acetonitrile-based mobile phases. PEA and OEA were detected by multiple reaction monitoring using the transitions m/z 300.1 → 62.0 and m/z 326.2 → 62.0, respectively.

### Statistical analysis

Data are presented as mean ± SD. The normality of the data distribution was assessed using the Shapiro–Wilk test, and homogeneity of variances was evaluated using Levene’s test. Statistical analyses were performed using GraphPad Prism 9.0. Comparisons among multiple groups were conducted by one-way ANOVA (Tukey’s test), while comparisons between two groups were performed using an unpaired Student’s *t* test. Statistical significance was defined as follows: ns: *p* > 0.05; ∗: *p* < 0.05; ∗∗: *p* < 0.01; ∗∗∗: *p* < 0.001.

Growth-related parameters were calculated as follows: survival rate (%) = (number of surviving fish/initial number of fish) × 100; weight gain rate ( %) = (final body weight − initial body weight)/initial body weight × 100; VSI (%) = (visceral weight/body weight) × 100.

## Data availability

The high-throughput sequences data reported in this study were archived in the Sequence Read Archive with the accession number PRJNA1474670. All other data needed to evaluate the conclusions in the paper are present in the article and the Supplementary Information.

## Supporting information

This article contains supporting information.

## Conflict of interest

All authors report no conflicts of interest in this work. All data were generated in-house, and no paper mill was used. All authors agree to be accountable for all aspects of work ensuring integrity and accuracy.
