# Supplementary material for: Hepatic stearoyl-CoA desaturase 1 suppresses diet-induced hyperphagia and hepatic lipid accumulation through PPARα activation and ketone body signaling
Source: J Biol Chem. 2026 Sep 22;302(10):113515. doi: 10.1016/j.jbc.2026.113515 (PMC13625769; doi:10.1016/j.jbc.2026.113515)
Supplement: Supplementary file 1 [file mmc1.docx]

**Supplementary materials**

**1. Supplementary figures and legends**

Figure. S1 Phylogenetic analysis and amino acid sequence alignment of SCD1 in representative cultured teleost species. (A) Phylogenetic tree of the SCD1 gene. (B) amino acid sequence alignment of SCD1.

Figure. S2 Validation of EGFP protein expression in liver-specific *scd1*-overexpressing zebrafish and lipid accumulation in zebrafish larvae after 7 days of feeding. (A) The EGFP and GAPDH expressions of the liver in *WT* and *scd1^+/+^* zebrafish using Western blotting. (B) TG, TC, and LDL-C levels in larvae. (C) Oil Red O staining of larvae. Numbers in the upper right corner indicate the percentage of larvae with the same phenotype. Scale bar = 500 μm.

Figure. S3 Validation of nuclear protein purity. GAPDH and Lamin B1 were detected by Western blotting in nuclear protein extracts from *WT* and *scd1^+/+^* zebrafish. GAPDH served as a cytoplasmic contamination marker, while Lamin B1 served as a nuclear marker/loading control. The absence or weak detection of GAPDH in nuclear extracts indicates minimal cytoplasmic contamination.

Figure. S4 Chromatograms of palmitoylethanolamide (PEA) and oleoylethanolamide (OEA) in liver and brain samples of adult zebrafish, as well as those of the two corresponding standards.

Figure. S5 The expression of appetite related genes and lipid accumulation in liver-specific *scd1*-overexpressing zebrafish larvae after 14 days of feeding. (A-B) the TG (A) and TC (B) contents in the WT zebrafish larvae after feeding ND, HFD, HFD+5 mg/kg LEI401, HFD+10 mg/kg LEI401, HFD+50 mg/kg LEI401, and HFD+100 mg/kg LEI401 for 14 days. (C) mRNA expression levels of *agrp*, *cart* and *gnrh2*. All values were normalised to *rps11* as internal reference.

Figure. S6 Hepatic level of NEFA in the liver of *WT* and *scd1^+/+^* zebrafish.

**2. Supplementary tables**

Table. S1 The list of primers used in this study.

| Purpose | Primers | Sequences (5’-3’) |
| --- | --- | --- |
| PCR | *scd1* *in-situ* fp | GGTGTATGGCCTCTTCCTGA |
|  | *scd1* *in-situ* rp | AGTCGTAGGGAAACGTGTGG |
|  | EGFP cds FP | ATGGTGAGCAAGGGCGAGG |
|  | EGFP cds RP | TTACTTGTACAGCTCGTCCATG |
|  | EGFP FP-lfabp10 pro | gtgttgatcgctaggggatccATGGTGAGCAAGGGCGAGG |
|  | EGFP RP OL | cagcctgcttcagcaggctgaagttagtagctccgcttccCTTGTACAGCTCGTCCATG |
|  | P2A | GGAAGCGGAGCTACTAACTTCAGCCTGCTGAAGCAGGCTGGAGACGTGGAGGAGAACCCTGGACCT |
|  | *scd1* cds FP | ATGCCTGATTCTGATGTGAAAG |
|  | *scd1* cds RP | TTAGCCGCTTCTGTAGCTGCC |
|  | *scd1* cds FP OL | cagcctgctgaagcaggctggagacgtggaggagaaccctggacctATGCCTGATTCTG |
|  | *scd1* cds RP OL | gctgattatgatctagagtcgcggccgcTTAGCCGCTTCTGTAGCTGCC |
|  | PPARα cas9 target site | taatacgactcactataggAGTGGAATATGCGCACCTCAgttttagagctagaaata |
|  | PPARα FP sanger | CATGACATGGAGACTCTTCAGC |
|  | PPARα RP sanger | ACGGCATTTATCAAATCGAATC |
| qPCR | q-*β-actin*-F | TGCTGTTTTCCCCTCCATTG |
|  | q-*β-actin*-R | TTCTGTCCCATGCCAACCA |
|  | q-*rps11*-F | ACAGAAATGCCCCTTCACTG |
|  | q-*rps11*-R | GCCTCTTCTCAAAACGGTTG |
|  | q-*srebp1*-F | GCTAGTTTTCATGGAATAACGC |
|  | q-*srebp1*-R | CATTGAGAAGGGCAGTGTCG |
|  | q-*pparg*-F | TCTCCGCTGATATGGTGGAC |
|  | q-*pparg*-R | GTCGATGCCTGATATGCTGC |
|  | q-*fasn*-F | GGAGCAGGCTGCCTCTGTGC |
|  | q-*fasn*-R | TTGCGGCCTGTCCCACTCCT |
|  | q- *ACC1*-F | TCAACTGCAGATCGCTATGG |
|  | q- *ACC1*-R | TCTGCAAACTCATGCAGTCC |
|  | q-*SCD1*-F | CTCTTCCTGATCCCGTCTGC |
|  | q-*SCD1*-R | GCCAGCCAACGTGAGAAAAG |
|  | q-*agrp*-F | CTGCCTGAAATGGAGCACCT |
|  | q-*agrp*-R | GGGCAGATGATGACCCAGAC |
|  | q-*cart1*-F | GTGGAGAAGAAGCTCGGCTGG |
|  | q-*cart1*-R | GCACTTGAGGATGGAGAAGCTGC |
|  | q-*cart2*-F | GGTCAAGTCCCCATGTGTGATTTGG |
|  | q-*cart2*-R | TCCTCAATCCCGTTACAATCCACTCC |
|  | q-*cart3*-F | GCTGTGCTGCTCTCCTGCATAC |
|  | q-*cart3*-R | GTAAAGCCCCGAGGAGCTGC |
|  | q-*cart4*-F | CGAGCAGGACGAGCAGTTCATC |
|  | q-*cart4*-R | GCTCCCCTACGTCACACCTG |
|  | q-*cnr1*-F | AGCGGAGCAGGACCAGTTCA |
|  | q-*cnr1*-R | GGGAACAGCATGGTCAGAGATGC |
|  | q-*galn*-F | GAGTTTGTGTGTCTCTCATTGTGTGCG |
|  | q-*galn*-R | CCATCCTCTCTTCTCTTTTGCCGC |
|  | q-*ghsra*-F | TGCCTGTGTTCTGCTTAACTGTCTTG |
|  | q-*ghsra*-R | CAAACACCACCACAGCCAGCA |
|  | q-*gnrh2*-F | ACATCCTCAAGACAATACTGCTGGA |
|  | q-*gnrh2*-R | GAAAAGGCAGGCCAAATGTG |
|  | q-*nmu*-F | CGACAGAGAGCATGAGGAACAGCA |
|  | q-*nmu*-R | GATTCAAGAGCATCGGAGCACTTGTG |
|  | q-*npy*-F | GACTCTCACAGAAGGGTATCC |
|  | q-*npy*-R | GGTTGATGTAGTGTCTTAGTGCTG |
|  | q-*nucb2a*-F | GCTGTGGAGCATATGGAGAGGCT |
|  | q-*nucb2a*-R | GGGAACTGGAGGCTGGTCTTG |
|  | q-*hcrt*-F | CTACGAGATGCTGTGCCGAG |
|  | q-*hcrt*-R | CCAAGAGTGAGAATCCCGAC |
|  | q-*pmch*-F | CTTCCCTTCAGAAGACACCCCATCA |
|  | q-*pmch*-R | TCCCTCCTTTAATTCCTGTGTCCGC |
|  | q-*pomca*-F | GCAAACGCAGACCCATCAAG |
|  | q-*pomca*-R | GTTATTTGCCAGCTCGCGTC |
|  | q-*pyy*-F | TGGGGATGACACAGAGCACAAAC |
|  | q-*pyy*-R | GGGAGGCACAGGTGGGTTTAG |
|  | q-*spx*-F | GAAAGGCACACAGGGACGGC |
|  | q-*spx*-R | TGGAGGATGTTCAGGAGAAACGC |
|  | q-*ehhadh*-F | GTCATGCCATCTCGAAGACCA |
|  | q-*ehhadh*-R | AACCCCTTCTATGGCAGCAA |
|  | q-*fabp1*-F | CACGACCGGATCAAAAGTGC |
|  | q-*fabp1*-R | TGATGCGCTTGTAGACGAGG |
|  | q-*hmgcs2*-F | TTCAATGCCGTCAACTGGGT |
|  | q-*hmgcs2*-R | CCACCACTGGATACTCCGAC |
|  | q-*abca1*-F | CCTCAGCATCTCCAGCTACG |
|  | q-*abca1*-R | AGCACCATCCAACCAGTCAG |
|  | q-*angptl4*-F | CTGGACAAACAGAACATCCGC |
|  | q-*angptl4*-R | TCGGGTGTCATCTCGCAGTA |
|  | q-*cyp7a1*-F | ACCTTCAACGAGCTGAGCAA |
|  | q-*cyp7a1*-R | TGTCCAACTGCTCCCTTGTC |
|  | q-*cyp8b1*-F | GCGAACACAAAGTTCTCCAAGC |
|  | q-*cyp8b1*-R | TGCCTTTGGGGGACTCATTAC |
|  | q-*abcb4*-F | CGCTGTCATCGGAAAGGTGA |
|  | q-*abcb4*-R | CAGGATAAGAGTGCTGCCGT |

**3. Original data of WB**
